# Harnessing Water to Enhance Quadrupolar NMR Spectroscopy and Imaging

**DOI:** 10.1002/chem.202201490

**Published:** 2022-09-26

**Authors:** Ricardo P. Martinho, Lucio Frydman

**Affiliations:** ^1^ Department of Chemical and Biological Physics Weizmann Institute of Science 7610001 Rehovot Israel

**Keywords:** chemical exchange, low-gamma MRI, quadrupolar NMR spectroscopy, sensitivity enhancement, water detection

## Abstract

^17^O and ^14^N are attractive targets for in vivo NMR spectroscopy and imaging, but low gyromagnetic ratios *γ* and fast spin relaxation complicate observations. This work explores indirect ways of detecting some of these sites with the help of proton‐detected double resonance techniques. As standard coherence transfer methods are of limited use for such indirect detection, alternative routes for probing the quadrupolar spectra on ^1^H were tested. These centered on modulating the broadening effects imparted onto protons adjacent to the low‐*γ* species through *J* couplings through either continuous wave or spin‐echo double‐resonance decoupling/recoupling sequences. As in all cases, the changes imparted by these double‐resonance strategies were small due to the fast relaxation undergone by the quadrupoles, the sensitivity of these approaches was amplified by transferring their effects onto the abundant water ^1^H signal. These amplifications were mediated by the spontaneous exchanges that the labile ^1^Hs bound to ^17^O or ^14^N undergo with the water protons. In experiments designed on the basis of double‐resonance spin echoes, these enhancements were imparted by looping the transverse encodings together with multiple longitudinal storage periods, leading to decoupling‐recoupling with exchange (D‐REX) sequences. In experiments designed on the basis of continuous on/off quadrupolar decoupling, these solvent exchanges were incorporated into chemical‐exchange saturation transfer schemes, leading to decoupling‐recoupling with saturation transfer (D‐REST) sequences. Both of these variants harnessed sizable proportions of the easily detectable water signals, in order to characterize the NMR spectra and/or to image with atomic‐site specificity the ^17^O and ^14^N species.

## Graphical abstract

^**17**^ **O and** ^**14**^ **N are attractive targets** for in vivo NMR imaging, but low gyromagnetic ratios *γ* and fast spin relaxation complicate observations. This work explores indirect ways to detect some of these sites with the help of double‐resonance techniques. These sequences ported the signatures of dilute, low‐*g* nuclei onto the abundant water ^1^H signal through the spontaneous exchanges that labile OH and NH protons undergo with H_2_O. These effects provide new routes in molecular NMR imaging.

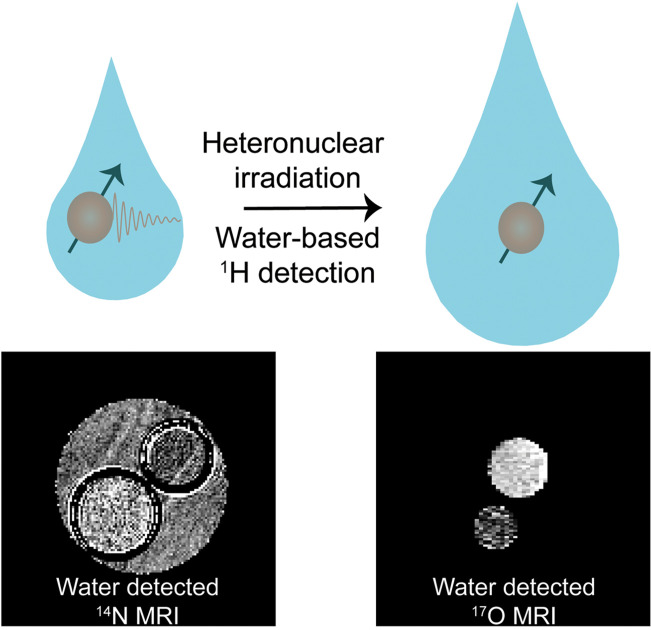

## Introduction

NMR spectroscopy and imaging (MRI) provide powerful non‐invasive routes to discern molecular signatures, both in vitro and when distributed throughout living organisms.[^1^, ^2^] Although numerous isotopes can be targeted by NMR/MRI most of them have low receptivity, making their observation difficult. One particularly important scenario where low sensitivity constrains a more widespread use of NMR arises for nuclides such as ^14^N, ^17^O or ^33^S, species characterized by nuclear spin *S*>1/2 , that are involved in numerous metabolic in vivo transformations. A common way to enhance the signal‐to‐noise ratio (SNR) of low‐sensitivity nuclides is by coherently transferring their spectral information to a directly bound ^1^H,^[3]^ whose high gyromagnetic ratio (*γ*) provides a more sensitive way to detect NMR signals. Transfers to/from *S*>1/2 nuclei, however, tend to be much less efficient than among spin‐1/2 counterparts,[^4^, ^5^, ^6^] as rapid *T*_2_ relaxation[^7^, ^8^] makes *J*‐based transfers inefficient[^9^, ^10^] (unless they involve quaternary amine sites with a high symmetry and a relatively slow ^14^N quadrupolar relaxation).[^11^, ^12^, ^13^, ^14^] Their quadrupolar nature also endows such nuclei with relatively fast *T*_1_ relaxation, which can be put to good use for extensive signal averaging and thereby SNR improvements.[^15^, ^16^, ^17^, ^18^, ^19^]

This work revisits the problem of enhancing the detection of quadrupolar nuclei bound to labile protons by exploring the possibility of imparting an amplified version of the low‐*γ* information onto the bulk water signal for a more sensitive NMR (and MRI) detection. Two routes are probed –both of them with roots in previous literature work. One is based on the seminal spin‐echo double‐resonance (SEDOR) experiment,[^11^, ^12^, ^20^] whereby an otherwise slow‐relaxing spin (the ^1^H) that is *J*‐coupled to a fast relaxing quadrupolar nucleus, will present altered *T*_2_ relaxation properties when subject to double‐resonance irradiation‐even in the absence of a resolved *J* splitting.^[21]^ This phenomenon, introduced in the 1960s by Meiboom,^[22]^ was used by Ronen et al. for imaging water's ^17^O in an indirect detection fashion.[^23^, ^24^, ^25^] The present study seeks an amplification of this effect by interspersing it with repeated magnetization storage periods, enabling an exchange between the labile, quadrupole‐encoding ^1^H, and the bulk water proton signal. A second route relies on double‐resonance techniques for modulating the longitudinal magnetization properties of the labile ^1^H based on the *S*‐spin characteristics. Such changes could then be passed on to the water ^1^H, in ways akin to those employed in chemical exchange saturation transfer (CEST).[^26^, ^27^, ^28^, ^29^, ^30^] As in the latter experiment, this results in an amplification of the spectral information of the quadrupolar nucleus, and in sufficient sensitivity to perform MRI. The principles and main features of these two approaches –which we denote decoupling‐recoupling with exchange (D‐REX) and decoupling‐recoupling with saturation transfer (D‐REST) in order to stress their relation to the frequency‐labeled by exchange (FLEX)[^31^, ^32^, ^33^] and CEST methods– are introduced in the next section. Thereafter we present results where the efficiency of these methods was used for mapping spectra and images of ^17^O, a 0.038 % natural abundance[^34^, ^35^] isotope of interest in imaging metabolic and functional processes,[^15^, ^36^, ^37^] as well for spectrally mapping and imaging various ^14^N‐containing biomolecules[^38^, ^39^, ^40^] and contrast agents.[^41^, ^42^] In all cases, this was achieved by observing solely the signal of bulk water.

### Theoretical background

**Figure 1:**
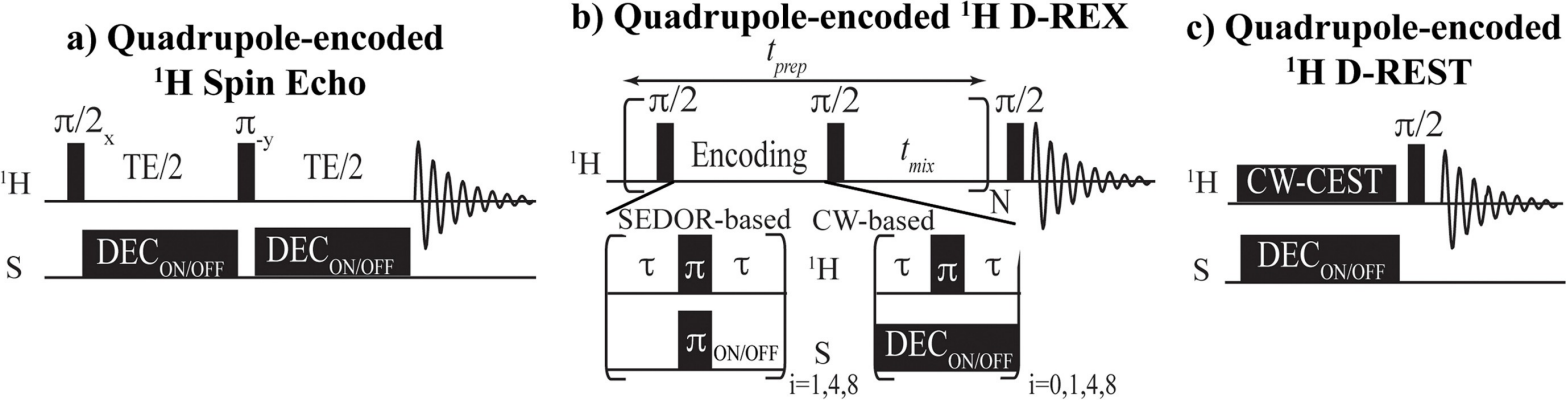
Pulse sequences considered in this work to highlight quadrupolar nuclei *S* bound to labile ^1^Hs on the water resonance. a) Spin echo (SE) scheme based on the work of Ronen et al.,^[23]^ in which *S*‐decoupling is/is not applied during an echo time TE and the difference on water is observed. b) D‐REX, a FLEX‐inspired scheme in which the *S*‐spin is/is not highlighted with either hard pulses or with *S*‐decoupling, and the whole module is repeated *N* times to port the *S*‐encoding thus imparted from the *S*‐bound proton to the water, as driven by exchanges happening during a mixing time *t*_mix_. *S*‐encoding modes were based on either SEDOR, that is, on applying a series of π pulses *i*=1 times (as in BIRD^[51]^), and *i*=4 or *i*=8 times using *xy*‐4 or *xy*‐8 phase cyclings.^[52]^ The *S*‐effects were also introduced by using a continuous decoupling (CW) scheme; this included an *i*=0 case representing a jump−return (JR) sequence^[53]^ in which *τ* comprises the full time in between π/2 pulses. c) D‐REST, a saturation transfer scheme in which an RF pulse is applied on protons for a selective saturation, while an *S*‐decoupling sequence is/is not applied on the *S*‐spin.

The scenario that is considered here involves a quadrupolar *S* nucleus (^17^O, ^14^N) that is directly bound to a proton, to which it is also *J*‐coupled. In general, the fast *T*_1_ relaxation of the quadrupolar nucleus will prevent the observation of resolved multiplets; still, the *S*‐spin will impart on the ^1^H an additional contribution to the transverse relaxation rate, by what is called scalar relaxation of the second kind^[43]^

(1) $$\frac{1}{T_{2,H}}=\frac{{\left(2\pi J\right)}^{2}S\left(S+1\right)}{3}T_{Q}$$

where *T*_Q_ is the quadrupolar *T*_1S_ relaxation time (in reality an approximation to it that is valid when *T*_1S_ ≈ *T*_2S_ ^[44]^), *S* is the quadrupole spin number, and *J* is the scalar coupling. Taking the ^17^O spins of water in vivo as an example, *T*_Q_ has been reported to be 4.47±0.14 ms,^[45]^ *S*=5/2 and *J* is 91 Hz;^[46]^ when assuming a fast exchange between the 3.8×10^−4^ proton population bonded to these ^17^Os at natural abundance with the protons in water (H_2_^16^O), this leads to an average broadening of the latter signal by ∼1.6 Hz. As for the ^14^N, it has a typical *T*_Q_ of ∼1 ms,^[47]^ *S*=1, and *J* of 62 Hz;^[48]^ when factoring its 99.6 % abundance, this leads to a ∼80 Hz broadening of the bonded proton. Regardless of their effective sizes, these broadening effects can be modulated by the application of an RF decoupling field on the *S*‐spin.[^44^, ^49^] If applying such field continuously and on‐resonance, the ^1^H broadening will be reduced to

(2) $$\frac{1}{T_{2,H}}=\frac{{\left(2\pi J\right)}^{2}S\left(S+1\right)}{3}\frac{T_{Q}}{1+{\left(\omega_{1}T_{Q}\right)}^{2}}$$

where *ω*_1_ is the strength of the applied *S*‐decoupling. This means that, in principle, it is possible to modulate this effect on/off and thereby highlight the *S*‐spin, by the presence/absence of the latter's decoupling. Kupce and Freeman took advantage of this effect for detecting ^17^O in water based on steady‐state acquisitions;^[50]^ Ronen et al. took this a step further, and adopted the scheme illustrated in Figure 1a to transform these effects into sensitive probes for imaging the presence of metabolic ^17^O.[^23^, ^24^, ^25^]

In fact, if the ^1^H involved is labile, both the magnitude of the broadening and therefore the feasibility of highlighting it by *S*‐spin decoupling, will also be affected by its chemical exchange with the water. The effects of solvent exchanges at an intermolecular exchange rate *k*_sw_ on the aforementioned transverse decoupled broadenings have also been investigated,[^22^, ^24^, ^25^] and transform Equation (2) into

(3) $$\frac{1}{T_{2,H}}=\frac{{\left(2\pi J\right)}^{2}S\left(S+1\right)}{3}\frac{\frac{\tau_{sw}T_{Q}}{T_{Q}+\tau_{sw}}}{1+({\frac{\tau_{sw}T_{Q}}{T_{Q}+\tau_{sw}}\omega_{1})}^{2}}$$

where *τ*_sw_=1/*k*_sw_. It follows from this that the aforementioned effects can be exploited as long as the quadrupole's relaxation time *T*_Q_≫*τ*_sw_. Eventually however, if the solvent exchange rate gets too fast, a “self‐decoupling” ends up erasing this relaxation contribution. If considering bulk water, for instance, all of the above‐mentioned effects will be attenuated by an intermolecular exchange rate *k*_sw_≈555 Hz under room temperature and neutral pH.^[22]^

A possible way to overcome the *T*_Q_‐ and *k*_sw_‐driven attenuations of the *J*‐driven broadening, is by extending the spin‐echo times; for instance for the case of water, a *TE* ≈1200 ms provides the scheme of Figure 1a with an observable contrast of about 10 % at natural abundance.^[23]^ Alternatively, overall SNR might improve if the effects of exchange are ported from the transverse ^1^H plane into a longitudinal axis that labels the ^1^H $\hat{z}\;$ magnetization. Figures 1b and 1c shows possible ways to do this, with *S*‐spin encodings inspired on SEDOR and CEST protocols, respectively. In all instances the *S*‐spins are targeted by a series of on/off experiments, from which differentials are taken on a readout focusing on the water resonance. The first of these experiments, decoupling‐recoupling with exchange (D‐REX, Figure 1b), introduces the *S*‐spin effects with on‐resonance pulses^[33]^ acting in unison with ^1^H spin‐echoing pulses. Following this *S*‐spin encoding the magnetization of the coupled ^1^H is stored along the z‐axis, and enabled to impart its information onto the water resonance during a period *t*_mix_. The whole scheme is then looped *N* times in order to magnify its effect up to the limit imposed by the water's relaxation time *T*_1,w_ ^[33]^ which, as previously derived for FLEX,[^32^, ^33^] can be expressed as

(4) $$S_{D-REX}=f\left[1-e^{-k_{sw}t_{mix}}\right]\times \sum_{i=1}^{N}e^{\left[-1+(i-1\right)/N]t_{prep}/T_{1,w}}e^{-(k_{sw}+{1/T}_{2,H})\tau }$$

where *i* corresponds to each individual loop of the experiment up to a total of *N*, *f* is the concentration of the exchanging solute, *t*_prep_ is the total preparation time, and the transverse relaxation of the labile proton *T*_2,H_ can be calculated in the presence/absence of exchange and decoupling using Equation (3).

**Figure 2:**
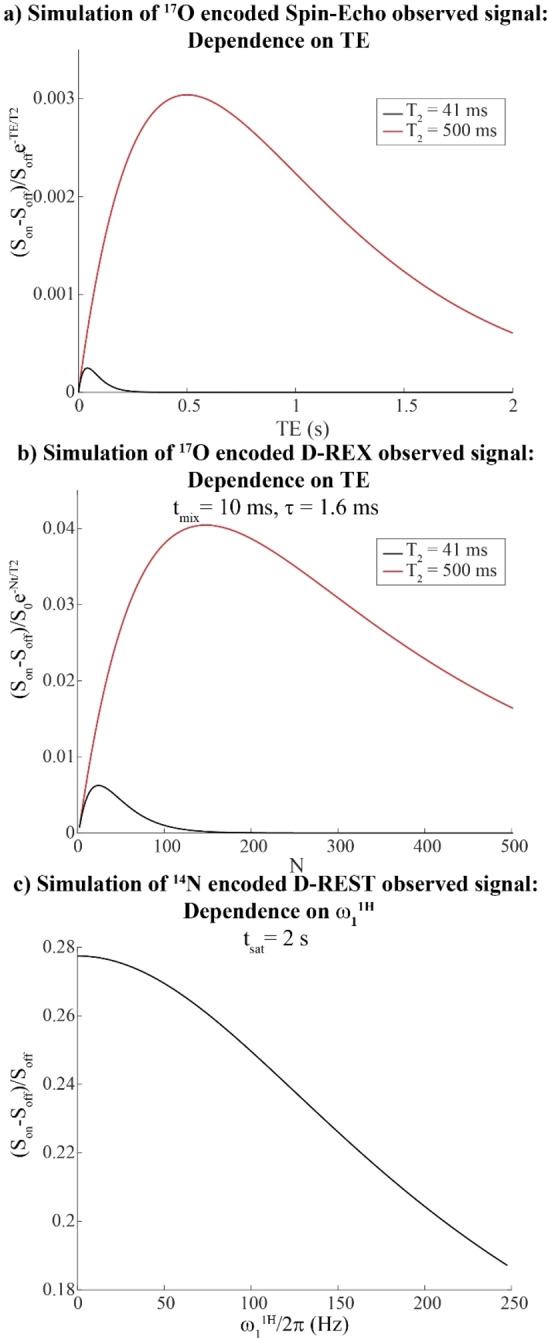
Analytical calculations of the signals resulting from the sequences introduced in Figure 1a, b for ^17^O in natural‐abundance water. *T*_Q_=4.4 ms,^[45]^ *J*=91 Hz,^[46]^ *k*_sw_=555 Hz,^[22]^ *T*_1,w_ 1.41 s, *T*_2_=41 ms (akin to gray matter^[54]^) or 500 ms (as in CSF). a) Simulation expected for the spin‐echo ^17^O‐encoded signal.^[25]^ b) Expectations for the ^17^O‐encoded D‐REX sequence and Equation (4), for *t*_mix_=10 ms, *τ*=1.6 ms, and optimization as in Figure S1. In (a) and (b), the responses account for the ^17^O natural abundance. c) Calculation of the D‐REST effect upon ^14^N‐encoding a dilute, 20 mM metabolite modeled on an amino acid,^[47,48]^ with an exchange rate of 200 Hz, *T*_Q_=1 ms, *J*=62 Hz, *T*_1,w_=1.41 s, *T*_2,H_=60 ms, 99.6 % natural abundance. The dependence of this effect on the proton saturation field for a 2 s long CW pulse was explored, and the effect was combined with the overall CEST effect.

Figures 2a and 2b compare the different performances expected from the sequences introduced in Figures 1a and 1b, for the case of natural abundance water that is self‐exchanging (see caption for parameters). For these estimations two different *T*_2_ values were taken for non‐^17^O‐bound water, akin to those in grey matter (41 ms) and CSF (500 ms), respectively, and the signal was weighted by the decay resulting from the extended encoding. For the quadrupole‐encoded spin‐echo experiment, the optimum sensitivity to the ^17^O is obtained for TE∼T2; for the *T*_2_=500 ms case, this would correspond to an ∼0.03 % change in the intensity of the initial water signal, which by now has decayed by ∼63 %. By contrast, it is possible to optimize the *τ*, *t*_mix_ and *N* values of the D‐REX strategy as shown in Supporting Figure S1, to achieve the larger ^17^O effects shown in Figure 2b. The increase in the ^17^O‐induced effects arises from the experiment's reliance on longitudinal storages that help preserve the water polarization. As shown in Figure S1, the ^17^O recoupling on this natural abundance sample reduces the water signal by ≈9 %; when taking into account the *T*_2_ times for water in CSF and white matter, this would be the equivalent to changes of 4 % and 0.6 % of the initial water signal intensity, respectively. These calculations also predict that the D‐REX approach can magnify ^17^O's presence by approximately 25‐fold versus the spin‐echo version for *T*_2_=41 ms, and by about 13‐fold for *T*_2_=500 ms. However, it is important to note that the repeated pulsing occurring in D‐REX can also result in further depletion of the signal as a result of experimental imperfections. Additional information concerning the D‐REX approach for different exchange and decoupling conditions are presented in Supporting Figure S2.

While both of these SEDOR‐based strategies show promise for enhancing the detection of quadrupolar nuclei, the short *T*_2_ values involved require the use of short *τ* values in order to impart significant enhancements. Therefore, these encodings are optimally performed with hard non‐selective pulses‐depriving the ^1^H dimension of spectral resolution. This is unimportant if focusing on ^17^O and bulk water; however, in some instances it would be desirable to perform frequency‐selective experiments leading to a 2D‐like encoding of the ^1^H and *S* resonances. To this effect we explored the D‐REST experiment shown in Figure 1c ‐a CEST‐related approach to enhance and map specific *S*‐spins, bound to specific labile protons. As in D‐REX, this method also aims at measuring all this information on the water resonance; this time as mediated by a CEST process targeting the labile ^1^H, whose efficiency will depend on whether this ^1^H is or is not *S‐*decoupled during its CW saturation. Under suitable conditions, introducing the decoupling should narrow the ^1^H line being saturated, thereby bringing a change in the amount of CEST effect being transferred to water. Care needs to be exercised here to ensure that the RF field applied on the ^1^H to implement its saturation does not become involved in some kind of *J*‐transfer to the *S*‐spin as driven by partially fulfilling a Hartmann‐Hahn match;^[44]^ in practice, CEST saturation fields were kept below the value needed to impart such effect (Figures S3a and S3b). In such instance, the magnitude of the effect can be estimated based on the analytical expression for the width of a peak being subject to D‐REST, which will be given by:^[55]^

(5) $$\nu_{1/2}=\sqrt{\omega_{1,CEST}^{2}\frac{p}{q}+p^{2}}$$

Here *ω*_1,CEST_ is the (continuous) RF field applied for the saturation ^1^H, $p=\;R_{2,H}+k_{sw}-\sqrt{\frac{k_{sw}k_{ws}}{R_{1,w}+k_{ws}}}$ , and $q=\;R_{1,H}+k_{sw}-\sqrt{\frac{k_{sw}k_{ws}}{R_{1,w}+k_{ws}}}$ , where *R*_2,H_, *R*_1,H_ and *R*_1,w_ are the transverse and longitudinal relaxation times of water (w) and of the labile (H) proton, and *k*_sw_/*k*_ws_ are the back and forth exchange rates between the labile (solute) and water ^1^Hs. *S*‐spin decoupling will affect the *R*_2,H_ term; these effects can be calculated from Equation (3), and used to assess the strength of the ensuing effect. Whereas applying this approach to water would result in direct saturation, and thus signal depletion, D‐REST can open doors to mapping other labile solutes‐for instance ^14^N‐bound protons such as amines, amides, etc. Figure 2c shows the performance that can be expected from this approach for a prototypical NH spin pair in an amino acid,[^47^, ^48^] with a moderate *k*_sw_ *=*200 Hz rate, *T*_Q_=1 ms, and *J*=62 Hz. While the ensuing effect on CEST will depend on the ^1^H saturating field employed, simulations predict an ^14^N‐imposed change of ≈3 % of the total water signal under these conditions ‐a change that should be measurable and thus open the route for ^14^N‐based detections. Notice that in this case the depletion of the water signal needs not be considered, as this is CEST's main observable.

## Experimental Section

The schemes introduced in the previous paragraph were explored on lysine, alanine, glycine, and urea‐all purchased at natural abundance from Sigma‐Aldrich. Samples were prepared in Dulbecco's phosphate buffer saline (PBS) with deuterated water (D_2_O), and their pH was adjusted with HCl as indicated in the captions. [D_6_]DMSO) was purchased from Merck, Fluorinert^TM^ FC‐770 from 3M^®^, and ^17^O‐enriched water from D‐Chem (Israel). A healthy female C57BL/6 mouse aged ≈6 months was used in an ex vivo experiment, preapproved by the Weizmann Institute's IACUC (#35520517‐2). The animal was sacrificed by cervical dislocation and its brain was removed and immediately placed in its entirety in Fluorinert^TM^ FC‐770 to prevent tissue modification. Unfortunately, not being fixed before its MRI scan, brain features were blurred in the ensuing images.

NMR data were acquired on a Varian V NMRS 300/89 (301.9 MHz for ^1^H, 40.9 MHz for ^17^O, 21.8 MHz for ^14^N) vertical bore 7 T magnet, equipped with a two channel 5 mm probe, and a two channel 10 mm probe compatible with a triple axis gradient system. Temperature was regulated with an FTS gas heater/chiller. All the sequences used herein were programmed and used as described in each caption, and can be obtained from the authors upon request. ^17^O and ^14^N π/2 pulses were measured to assess the necessary RF field for decoupling, being ∼12 and ∼30 μs in the 10 mm probe, respectively. Their spectra were externally referenced in relation to 1,4‐dioxane^[34]^ and ammonium chloride,^[38]^ respectively. Typical “off” data were collected by setting the decoupling frequency 10 kHz away from the on‐resonance position, to ensure that similar power depositions onto the sample as in the “on” cases were kept. Standard ^1^H acquisitions were employed to readout the different water‐detected experiments, using π/2 pulses of ∼24 μs, a 6000 Hz spectral width, 32 768 complex points, and 5 s recycle delays. Time‐domain signals were Fourier transformed, phased, and their water resonances integrated in Matlab for all of the methods here described. Imaging experiments were performed using the scanner's RARE implementation, coupled with the required encoding. All images were reconstructed using Matlab through 2D FT, and difference imaging was performed by subtracting the two differently‐encoded images acquired. The custom codes used to perform this processing and other calculations described herein, are also available upon request.

## Results

### D‐REX ^17^O NMR and MRI

**Figure 3:**
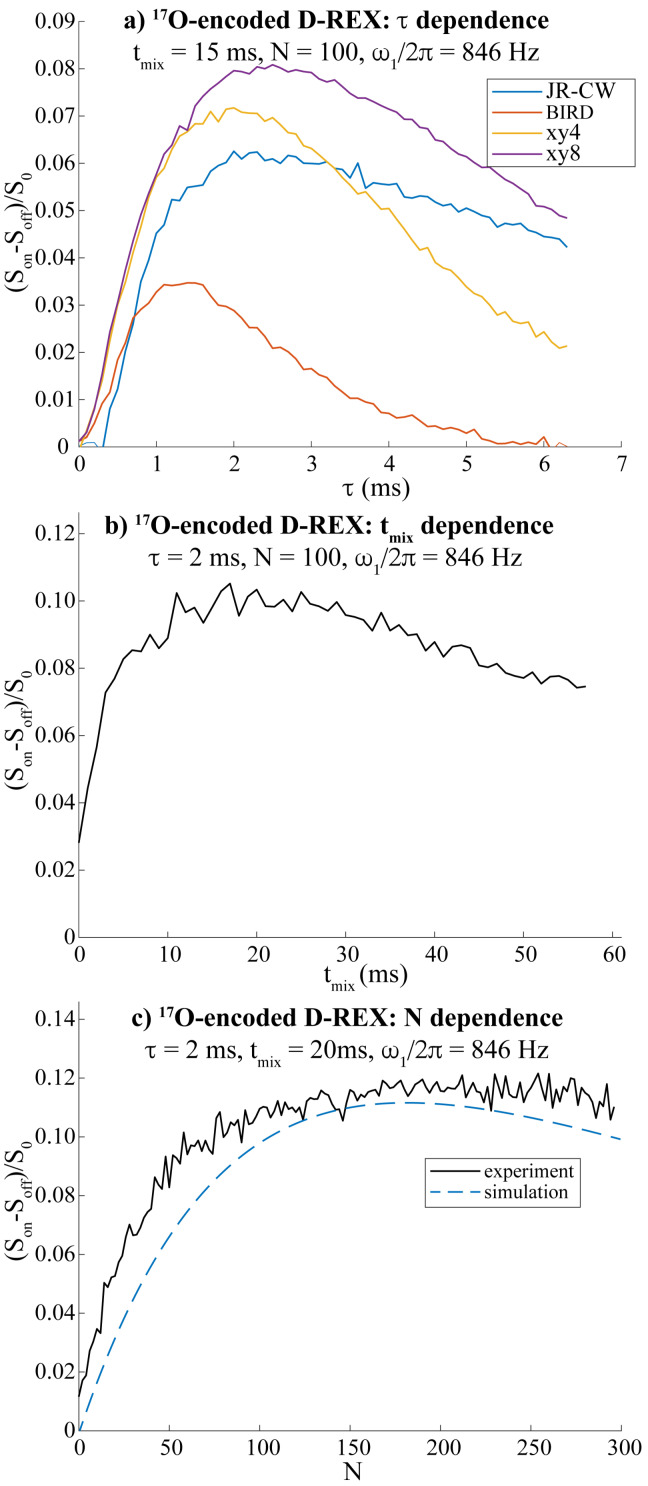
Optimization of the different parameters regulating ^17^O‐encoded D‐REX, conducted on a 50 : 50 mixture of water and [D_6_]DMSO where water was enriched with 25 % ^17^O. All the experiments were acquired at 7 T and 24 °C with an ^17^O RF field of 846 Hz. a) Optimization of *τ*, with *N*=100, *t*_mix_=15 ms, for the different D‐REX modes described. b) Optimization of *t*_mix_, with *τ*=2 ms, and *N*=100. A *t*_mix_ of 20 ms was estimated as optimal. c) Optimization of *N*, with *t*_mix_=20 ms and *τ*=2 ms. b) and c) were run with the JR‐based D‐REX, which in the best case achieved an ∼11 % contrast on the ^1^H water signal. Also included in this panel is a simulation based on water's H_2_^17^O quadrupolar parameters, an exchange rate *k*_sw_=50 Hz, *T*_1,w_=2 s, *T*_2,H_=0.65 s, and other parameters as in the experiment itself.

As a first test of the alternatives described in the theoretical background, a series of ^17^O‐encoded D‐REX experiments were run using ^17^O‐enriched water mixed with DMSO. ^17^O‐enrichment eased the search for optimal calibration parameters, while providing a more reliable performance estimate. The sequence was optimized along the lines of the simulations shown in Supporting Figure S1; that is, by varying the recoupling time *τ*, the exchange time *t*_mix_ and the number of loops *N*; also a variety of schemes for decoupling/recoupling the ^17^O were tested. A summary of these results is presented in Figure 3. Figure 3a shows the magnitude of the effects for four different encoding modes, as a function of recoupling time *τ*. The optimal *S*‐irradiation scheme for these conditions was found in an xy‐8 CPMG loop,^[52]^ closely followed by an *xy*‐4 one‐probably because these are likely to handle RF inhomogeneities better. (Further evidence for this can be seen in Supporting Figure S4, where shimmed and unshimmed versions of these experiments are compared‐and again *xy*‐based versions provide the best performances). In most instances the experimentally observed optimal *τ* was ≈2 ms; this differs from the 1.2 ms theoretically expected to maximize the coherence transfer to a spin‐5/2 coupled by a *J*=91 Hz,^[4]^ probably reflecting the recoupling changes introduced by the ^17^O relaxation and exchange averaging effects; similar observations have been made for ^1^H,^17^O 2D correlation experiments.^[10]^ Maximizing the D‐REX benefits then requires repeating this process at *t*_mix_≈1/*k*_sw_ intervals, and looping it up to *N*⋅*t*_mix_ *≈T*_1,w_ (Figure 3b, c). The resulting effect ends up reducing the water resonance by ∼11 % ‐a number that is on the order of the ^17^O concentration for this sample, as expected given the relatively slow exchange rate in this DMSO‐based sample (see figure caption). It should be noted however, that this 11 % is measured against a reference signal *S*_o_, that has decayed to about 70 % of the original, untouched ^1^H water resonance as a result of the manipulations involved in the D‐REX encoding.

**Figure 4:**
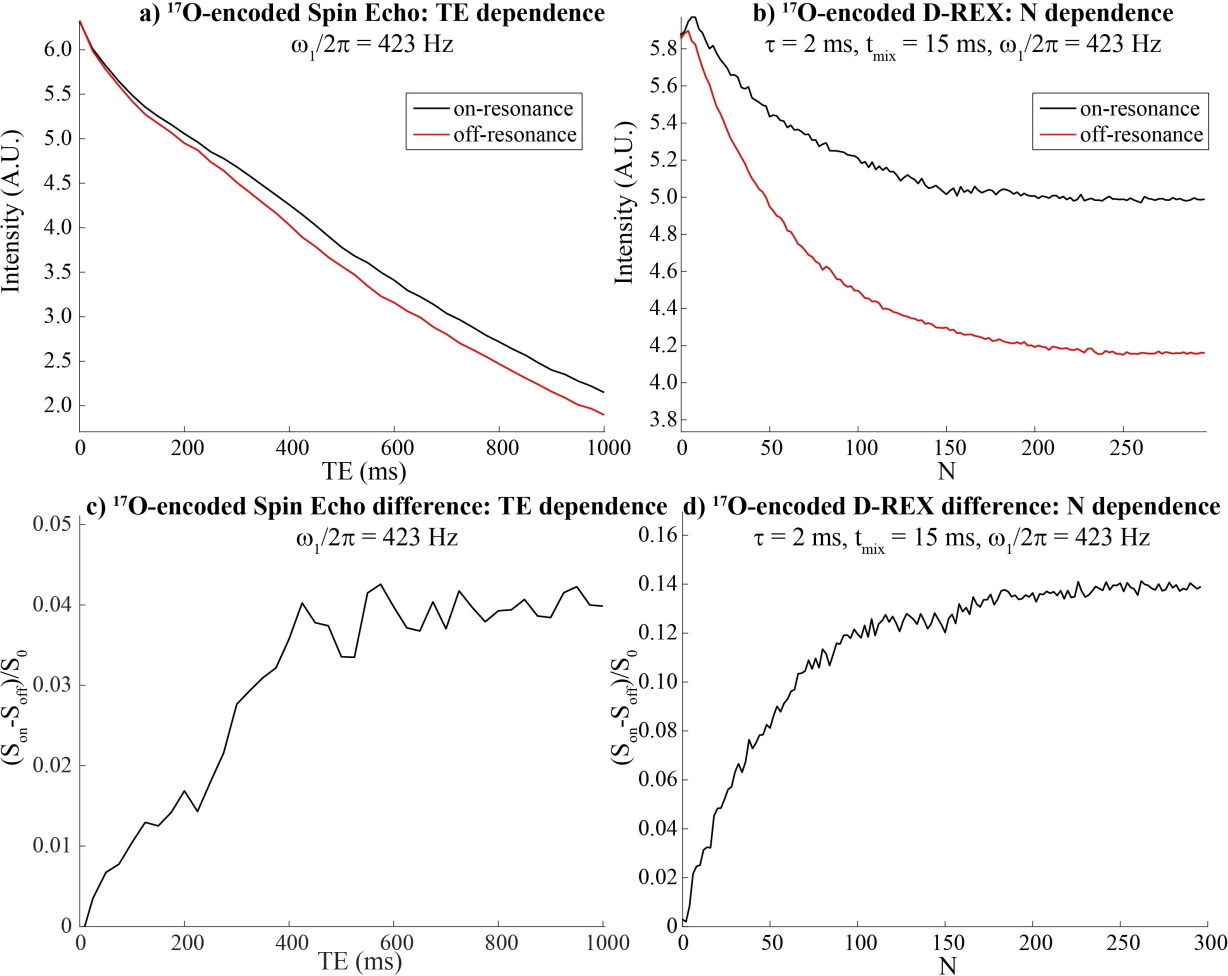
Comparisons between the enhancements afforded by ^17^O‐encoded SE experiments at different echo times TE (a and c), and by ^17^O‐encoded D‐REX for different *N* loop numbers (b and d). For the latter, a JR version of D‐REX was employed. Experiments were conducted on the same sample as in Figure 3, at 7 T and 37 °C with the indicated parameters.

Figure 4 presents other aspects of these methods, by comparing the ability of an optimized D‐REX acquisition to detect water's ^17^O, against a spin‐echo counterpart (sequence in Figure 1a). Both experiments lead to an unwanted decay of the water signal: in the latter due to transverse relaxation, and in the D‐REX as a result of relaxation plus the effects of the cumulative pulse imperfections (Figures 4a, 4b). Still, in the range of the useful, plateauing ^17^O‐encoding (≈600 ms for the spin‐echo, ≈150 loops for the D‐REX), the ratio of the water signal preserved in these experiments is 45 % and 70 % respectively. While as shown in Figures 4c and 4d the D‐REX‐based encoding leads to a ≈3.5x higher sensitivity contrast for the detection of ^17^O in water than its spin‐echo counterpart, this enhancement is substantially smaller (≈30 %) than what is predicted based on the aforementioned analytical expressions. We ascribe this lack in efficiency to the cumulative effect of pulse imperfections during the repeated storages of the refocused magnetization.

**Figure 5:**
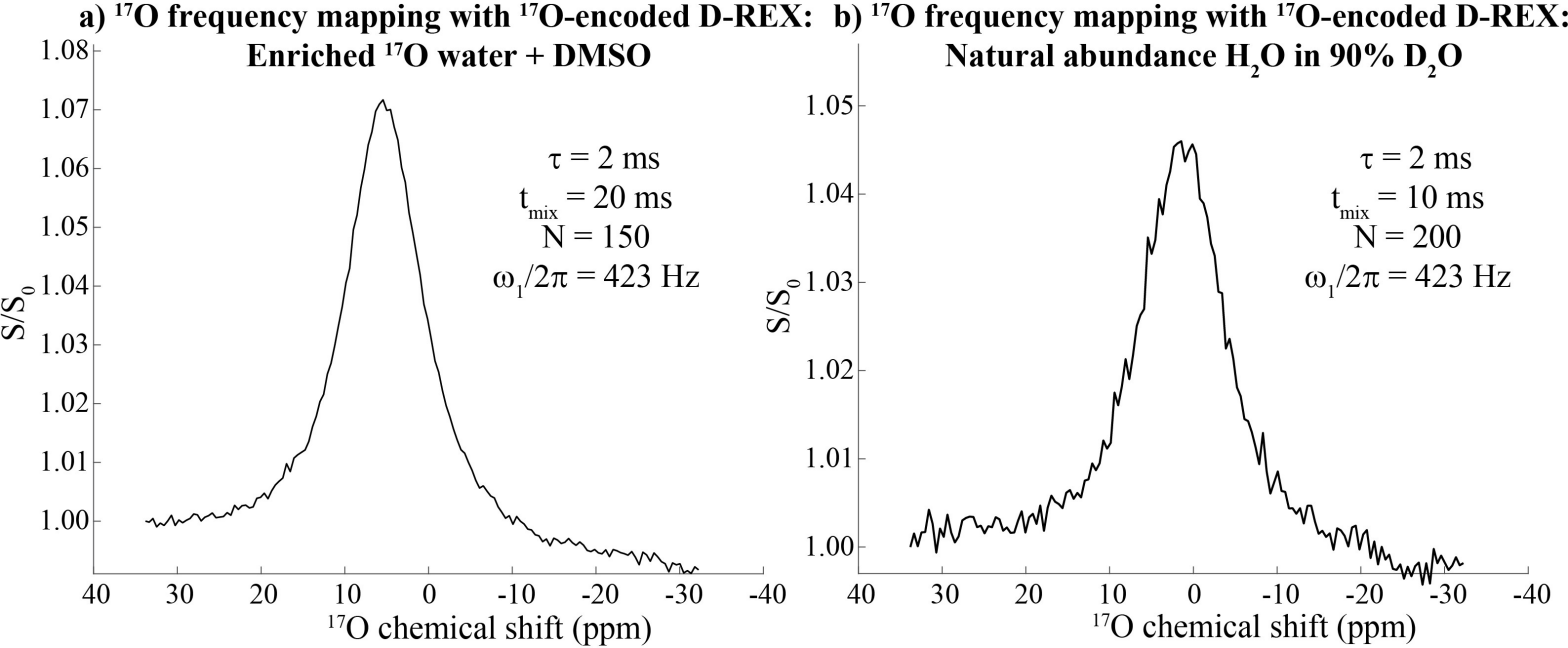
Indirect chemical shift mapping of the ^17^O NMR spectrum for a) the sample introduced in Figure 3 and b) 10 % natural abundance water in 90 % D_2_O. Both spectra involved a point‐by‐point scanning of the ^17^O offset over 70 ppm in increments of 1 ppm, where each point corresponds to one scan, normalized by the first point (which is considered as off‐resonance for ^17^O). A JR ^17^O‐encoded D‐REX sequence was used in both cases with the indicated parameters, in ∼10 min each (7 T field, 24 °C).

The contrast provided by these experiments can be employed to map an ^17^O NMR spectrum. As D‐REX's encoding marks the presence of ^17^O but not its resonance frequency, the latter can be reintroduced by stepping, in a point‐by‐point fashion, the offset frequency of the decoupling pulses encoding the *S*‐spin. Although this is reminiscent of spin tickling double resonance CW NMR experiments,^[56]^ each of these data points is now acquired in a single scan. In approximately 10 minutes and with ∼5–7 % contrasts, one can thus map both ^17^O‐enriched as well as natural abundance water on the ^1^H_2_O signal. This contrast is presented in Figure 5; notice that the D‐REX contrasts are not significantly different for the natural abundance and for the enriched samples, as a result of the higher exchange rates favoring the enhancement in the former case. Increasing the strength of the RF fields employed to encode the ^17^O will reduce the effective spectral resolution (Supporting Figure S5), but may also lead to a much more rapid scanning of the spectrum being sought. This could be of use in scanning natural abundance metabolites possessing relatively broad ^17^O lines.

**Figure 6:**
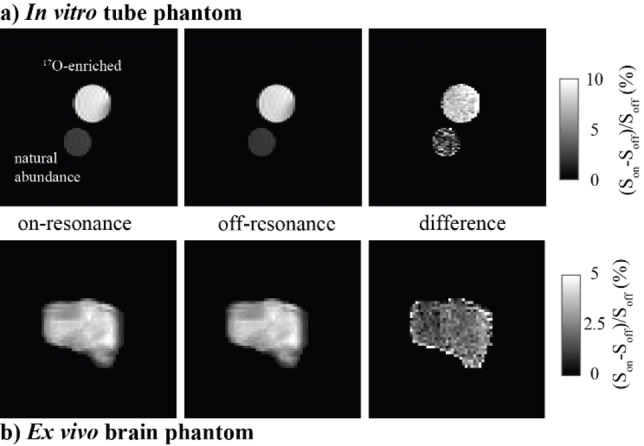
Images resulting from *xy‐*8‐based ^17^O‐encoded D‐REX, with the final pulse in Figure 1b replaced by a RARE detection (RARE‐factor=8). a) Experiments performed on the sample introduced in Figure 3, with *τ*=4 ms, *t*_mix_=20 ms, *N*=80, slice thickness=3 mm, in‐plane resolution=150x300 μm^2^, 64 phase encodes, 1 scan per phase encode, acquisition time of 3 min 12 s. b) Experiments on an extracted brain immersed in Fluorinert, conducted with *τ*=0.6 ms, *t*_mix_=20 ms, *N*=40, slice thickness=5 mm, in‐plane resolution=313x313 μm^2^, 64 phase encodes, 8 scans per phase encode, acquisition time of 18 min 8 s. Both experiments employed a decoupling field of 424 Hz and were performed at 7 T and 24 °C.

By affecting a reasonable percent of the water ^1^Hs, D‐REX sensitivity may be sufficient to allow some forms of ^17^O imaging. To explore this option, ^17^O‐encoded D‐REX was coupled to a RARE imaging sequence, and their potential was assessed on the two samples shown in Figure 6. For liquid tubes, D‐REX provided images with a considerably high resolution‐much less blurred than what common methods would furnish on ^17^O; sensitivity was also high, with approximately 8 % of the water signal marking the ^17^O for the enriched sample. These experiments were also tested on an ex vivo brain, to demonstrate signal could be obtained from it. The water contrast provided by this experiment amounted to ∼1–2 % (Figure 6b)‐this is in line with the analytical expectations presented in Figure 2. This suggests that despite the relaxation/exchange times arising in tissues, and despite the potential effects other macromolecules present in tissue, D‐REX‐based ^17^O imaging can be performed. Moreover, since most applications of these method would occur in conjunction with some ^17^O enrichment by inhalation or injection, SNR can likely be higher and therefore translatable into in vivo studies.

**Figure 7:**
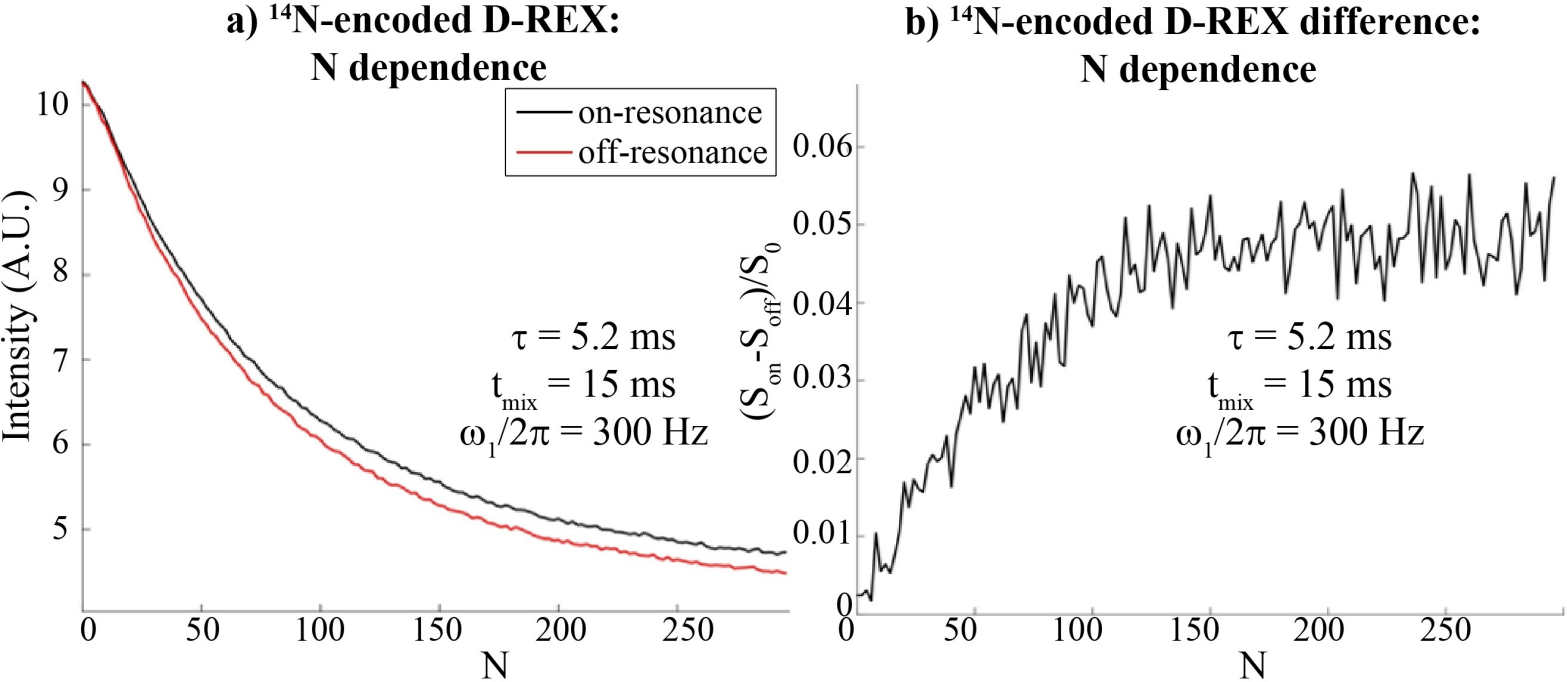
^14^N‐encoded D‐REX experiments on a 1.86 M, pH 1 glycine solution in 10 % D_2_O at 7 T and 24 °C, with a JR‐CW version of D‐REX. *N* was optimized with on‐resonance decoupling at 10 ppm with a field of 300 Hz; a) the two individual spectra and b) the difference. Experiments were performed with optimized *t*_mix_=15 ms and *τ*=5.2 ms, with a ∼5 % contrast on the ^1^H water signal being observed.

In order to explore the applicability of this approach to other quadrupolar nuclei, D‐REX was assayed for the ^14^N encoding of a 1.86 M glycine solution (Figure 7). The method yielded a ∼5 % contrast on water, which given the water's decay translates into an ∼2.5 % initial water signal intensity effect. In an effort to achieve some spectral resolution these D‐REX experiments were performed with jump‐return (JR) encoding (Figure 1b); although this preserved the ^1^H chemical shift information, it also resulted in losses given the longer encoding times required by this procedure.

### D‐REST ^14^N NMR and MRI

**Figure 8:**
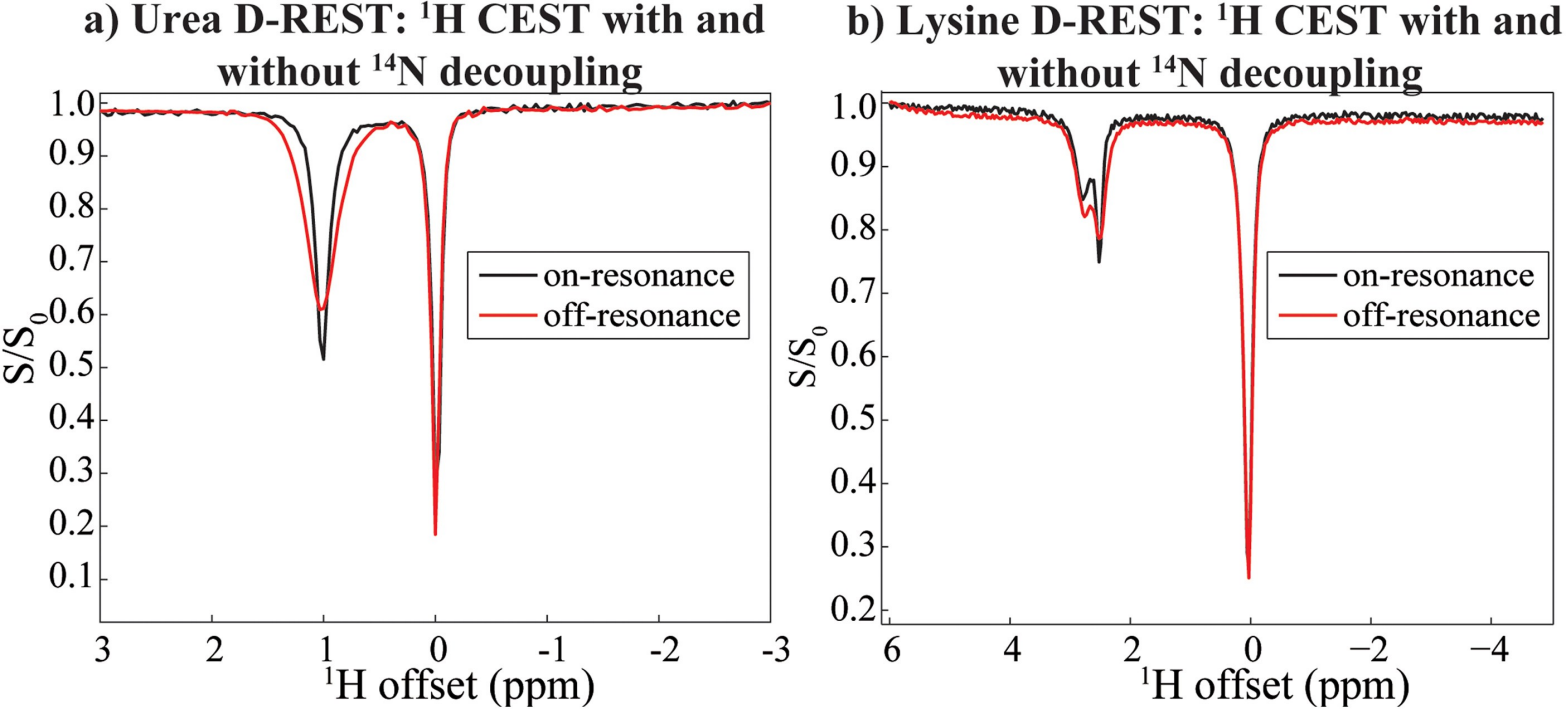
D‐REST experiments detecting a low‐*γ* ^14^N nucleus on the abundant ^1^H signal of water (set to 0 ppm). a) Comparison of on‐ and off‐resonance CW decoupling on the CEST spectral mapping of a 200 mM urea sample at pH=7.1; an RF field of 10 Hz was applied for 1 s for the proton saturation. The on and off experiments involved ^14^N decoupling at 0 and at 450 ppm from the urea ^14^N resonance, respectively. b) Idem for a 1 M lysine sample at pH 2.0, with a proton ω_1_ of 10 Hz applied for 1 s for saturation, and ^14^N decoupling applied at 21 and 471 ppm for the on/off experiments (lysine's ^14^N^ϵ^ resonating was the only one identified at 20.6 ppm) All experiments were performed at 7 T and 24 °C and employed decoupling nutation fields of 1.4 kHz, 1 scan per ^1^H offset, and a ^1^H offset incremented in 0.1 ppm steps.

D‐REX manages to map and image quadrupolar species like ^17^O thanks to a number of assisting factors including *J*‐couplings and fast exchanges with the water; nevertheless, it hides a proton chemical shift information that could become informative. ^14^N characterizations‐aided by a high natural abundance but hurt by low in vivo concentrations, by potentially fast quadrupolar relaxation, and by a wide range of exchange rates for labile amide and amine protons‐raise a challenge that may benefit from a different approach. Indeed, rapid *S*‐spin relaxation leading to self‐decoupling, and ^1^H resonances broadened by both exchange with the water and by scalar relaxation, may end up requiring longer D‐REX evolution times to monitor the presence of recoupling, thereby suffering from more severe SNR losses. In order to permit these processes to act over long periods of time without such penalties we also assayed the experiment introduced in Figure 1c, which measures CEST Z‐spectra of labile protons in the presence and absence of *S*‐spin irradiation. Figure 8a shows the results of such a D‐REST experiment, as applied for encoding the ^14^N of natural abundance urea dissolved in water. When executed with the provisions described in the theoretical background‐that is, weak ^1^H saturation fields and strong ^14^N decoupling fields‐this experiment resulted in a noticeable line narrowing of urea's ^1^H resonance (not shown), that then reflects on a ≈15 % change in the water‐detected CEST response of urea. Figure 8b shows the more complex behavior observed when the same scheme is applied on lysine, a molecule possessing two kinds exchangeable of protons bound to different kind of ^14^Ns: a broader ^1^H resonance around 3 ppm downfield from water arising from the NH_2_^α^, and a narrower ^1^H about 2.6 ppm downfield from water arising from the NH_2_^ϵ^. Upon introducing ^14^N decoupling at 21 ppm (i. e., close to the NH_2_^ϵ 14^N, the only one being observed^[38]^), NH_2_^ϵ^ shows the expected narrowing, while the latter barely changes; in fact, the NH_2_^α^ CEST response seemingly decreases, due to losing part of the overlap it exhibited with the broader NH_2_^ϵ^ peak. Still, in unison, these changes lead to an approximately +10 % signal change compared with the same CEST experiment performed without decoupling.

**Figure 9:**
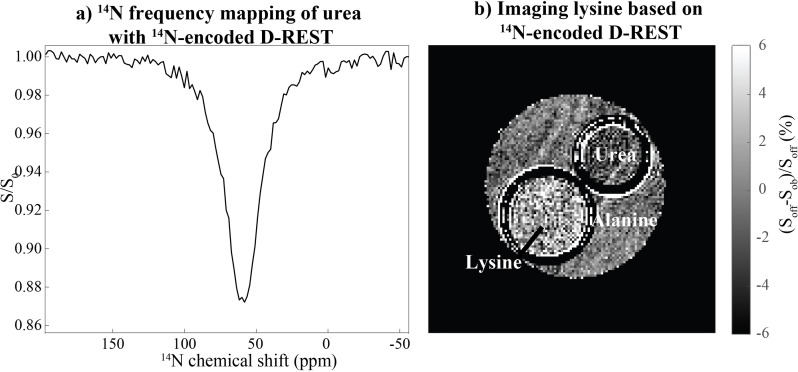
a) ^14^N chemical shift mapping in the urea sample introduced in Figure 8, arising from stepping the ^14^N decoupling frequency in a D‐REST experiment over 250 ppm in 2 ppm steps. For all steps, the urea protons were saturated with a 10 Hz field at +1 ppm downfield from water, and a ^1^H water spectrum was collected thereafter. b) D‐REST experiment targeting a urea/lysine/alanine phantom, incorporating a RARE block as signal acquisition. Lysine and urea were as in Figure 8; alanine was present at 100 mM and pH 2.2. The NH_2_^ϵ 1^H resonance of lysine was saturated with a field of 16 Hz on resonance at +2.6 ppm, and performed as a difference experiment between ^14^N on‐resonance decoupling at 28.8 ppm and an off‐resonance decoupling spectrum, slice thickness=2 mm, in‐plane resolution=117×117 μm^2^, 128 phase encodes, 1 scan per phase encode, RARE factor of 8, acquisition time of 1 min 4 s. All remaining conditions were as in Figure 8.

Like D‐REX, D‐REST can also be used to map an ^14^N chemical shift spectrum by keeping fixed the CEST saturating field on the labile ^1^H resonance, and stepping over the ^14^N decoupling frequency. Figure 9a shows how changes in the water signal intensity will reflect such spectral acquisition for a urea solution. Given the water‐based sensitivity of this D‐REST spectral encoding, its realization can also be extended onto an imaging mode‐without giving up the ^14^N spectral selectivity given by the decoupling offset. This is demonstrated in Figure 9b, where a phantom containing a variety of ^14^N‐bound labile protons can yield lysine‐specific images, by suitable combination of the ^1^H saturation and of the ^14^N decoupling resonances. These images resulted from subtracting two identical RARE scans, with on‐ and off‐resonance ^14^N decoupling; being relatively fast, the sensitivity of these spectral images could naturally be improved by signal averaging.

## Discussion and Conclusions

Methods for enhancing the detection of quadrupolar nuclei bound to labile protons, aimed in particular at biological translation, were introduced and demonstrated on different samples. As coherence transfer methods are difficult to realize when fast relaxing nuclei are bound to labile protons, here the information from the quadrupolar nucleus was sought by combining two phenomena: i) altering the residual broadening imparted to the quadrupolar‐bound proton through partially averaged scalar *J*‐couplings by using differential decoupling/recoupling procedures, and ii) amplifying these differential broadening effects by exchange‐driven saturation transfer processes, which ended up affecting sizable proportions of the water magnetization. Ultimately, it was the latter that reported on the quadrupoles‐both as spectra and as images. An important shared aspect of the proposed experiments was imparting the *J*‐related information to amplitude modulations of the labile ^1^Hs’ longitudinal magnetization. In the case of ^17^O, where the residual broadenings were smaller, pulsed methods incorporating short periods of transverse evolution with/without decoupling, looped together with longitudinal mixing periods introduced for facilitating exchanges with the rest of the solvent, led to a variety of so‐called D‐REX experiments. The enhancement of this method in comparison to direct detection can be qualitatively gauged based on the differences between the gyromagnetic ratios *γ* of the observed nuclei, the magnifying effects of the chemical exchange, the *T*_2_‐driven signal decay involved in the indirect detection, and the different recovery times (*T*_R_) expected from significantly different ^1^H and quadrupolar *T*_1_ values, leading to different averages being acquired. Factoring in all of these NMR signal dependencies and noise‐reducing factors leads to an expected sensitivity enhancement of

(6) $${D-REX}^{enh}\approx \frac{\frac{\gamma_{H}^{2}}{\gamma_{S}^{2}}\times k_{sw}T_{1,w}\times e^{-N\tau /T_{2}}}{\sqrt{\frac{T_{R,H}}{T_{R,S}}}}$$

For the aforementioned exchange rates and relaxation times, a ratio of *T*_R_ where pulsing on the quadrupole is ≈100× faster than on its ^1^H counterpart, and a *T*_2_‐driven signal decay over the course of the looping of 50 %, Equation (6) leads to a D‐REX enhancement of ∼1000× relative to direct detection for ^17^O. The approach thus leads to a larger contrast than previously discussed ^17^O‐encoding procedures based solely on transverse spin echoes;[^23^, ^24^, ^25^, ^57^, ^58^, ^59^] although applied here for the detection of self‐exchanges within water, applications to hydroxy groups‐arising for example from ^17^O‐glucose‐can also be envisioned.

A related but different approach was employed to enhance the water detection of ^14^Ns adjacent to labile protons. Here the route taken involved modulating the linewidths of the labile ^1^Hs, and detecting them through CEST‐based water NMR/MRI. This modulation was implemented by ^14^N decoupling. When implemented on an imaging mode, this D‐REST strategy required addressing specific ^1^H and ^14^N chemical shifts, thus yielding the pseudo‐2D NMR spectral editing that we sought. This in turn opens up interesting routes for imparting specificity into CEST‐based methods, which suffer from significant overlap between the many metabolites present.[^60^, ^61^, ^62^] Following a similar analysis as that leading to Equation (6), the aforementioned conditions translate into an optimal enhancement of ∼2000× in relation to directly detected ^14^N signal. Still, for D‐REST to be effective, a number of conditions need to be met: quadrupolar relaxation needs to be moderate as otherwise the *J*‐related effects will become too small and ^14^N decoupling inconsequential, and the labile ^1^H has to be under sufficiently slow exchange to prevent self‐decoupling, but fast enough to provide sufficient CEST enhancement onto the water signal. It remains to be seen how often this is met in relevant in vivo scenarios. Another complication of D‐REST over D‐REX is the need to apply relatively long periods of heteronuclear decoupling; although requiring RF fields of only a few 100 Hz, these may be challenging to achieve even on animal‐oriented scanners without too much power deposition. Tailored decoupling sequences could facilitate this. The main advantage of this method for targeting amines and amides, in comparison to D‐REX, is the fact that both ^1^H and ^14^N chemical shifts are targeted, thus providing further chemical specificity.

## Conflict of interest

The authors declare no conflict of interest.

1

## Supporting information

As a service to our authors and readers, this journal provides supporting information supplied by the authors. Such materials are peer reviewed and may be re‐organized for online delivery, but are not copy‐edited or typeset. Technical support issues arising from supporting information (other than missing files) should be addressed to the authors.

Supporting Information

## Data Availability

The data that support the findings of this study are available from the corresponding author upon reasonable request.
