# Supplementary material for: Harnessing Water to Enhance Quadrupolar NMR Spectroscopy and Imaging
Source: Chemistry. 2022 Sep 26;28(58):e202201490. doi: 10.1002/chem.202201490 (PMC9828088; doi:10.1002/chem.202201490)
Supplement: Supplementary file 1 — Supporting Information [file CHEM-28-0-s001.pdf]

Chemistry–A European Journal

Supporting Information

Harnessing Water to Enhance Quadrupolar NMR Spectroscopy and Imaging

Ricardo P. Martinho and Lucio Frydman*

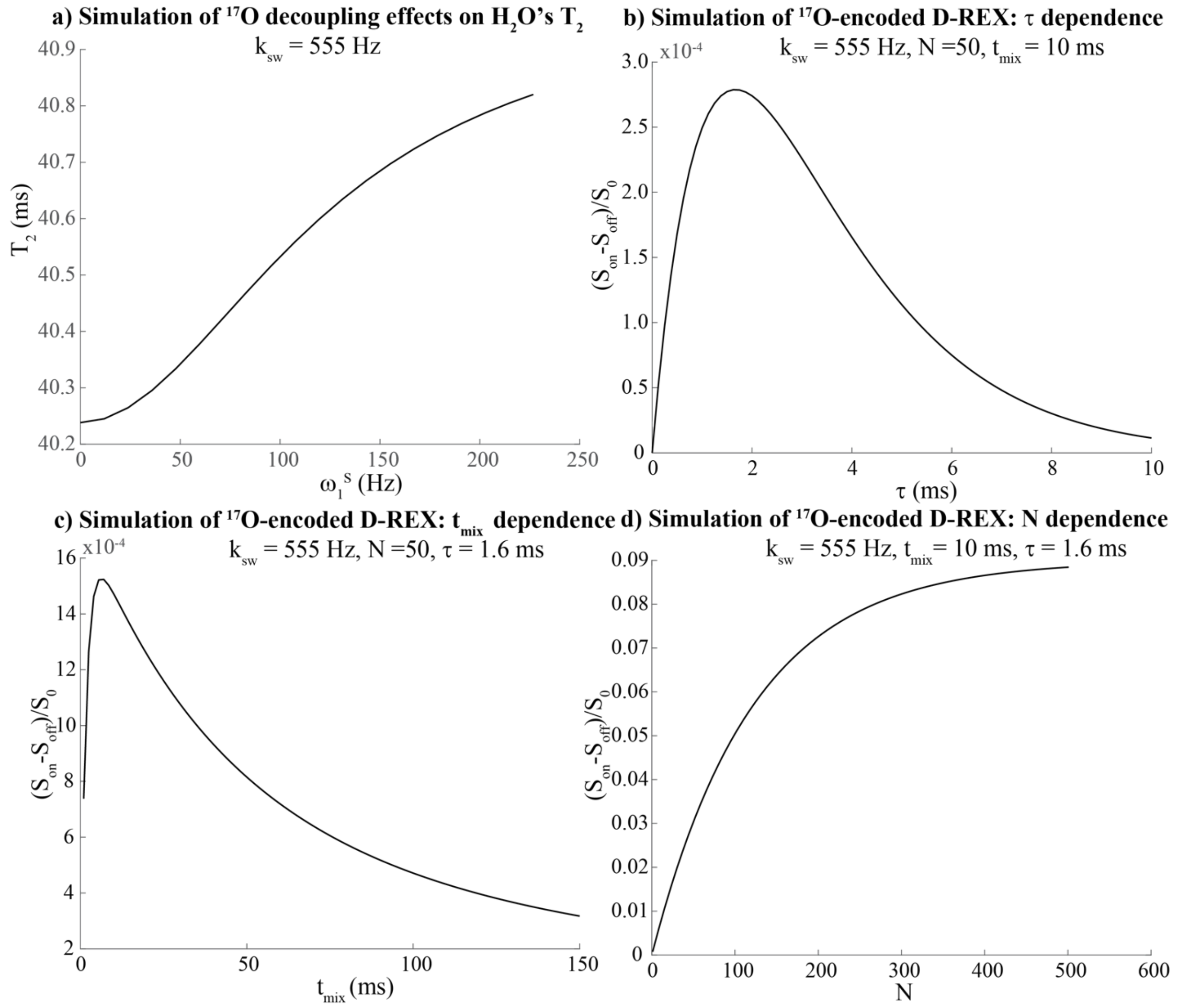

Figure S1: Analytical calculations of the effects of ^{17}O decoupling in natural abundance water, taking T_Q = 4.4 ms,[1] J = 91 Hz,[2], k_{ex} = 555 Hz,[3] $T_{1,w}$ = 1.41 s, and the natural $T_{2,H}$ as 41 ms. a) Effect of decoupling into the average water T_2 with different decoupling fields, based on equation (3). b-d) Calculations of the ^{17}O-encoded D-REX effects based on equation (4). b) Calculation of the magnitude of the effect with regards to τ, with N = 50, and a t_{mix} of 10 ms. The optimal value was 1.6 ms and used henceforth. c) Calculation of the magnitude of the effect with regards to t_{mix}, with τ of 1.6 ms, and N = 50. *A* t_{mix} of 10 ms was estimated as the optimal. d) Calculation of the magnitude of the effect with regards to N, with t_{mix} of 10 ms, and τ of 1.6 ms. The optimal N is ~ 350 and this a maximum enhancement of ~ 9% of the water signal is predicted.

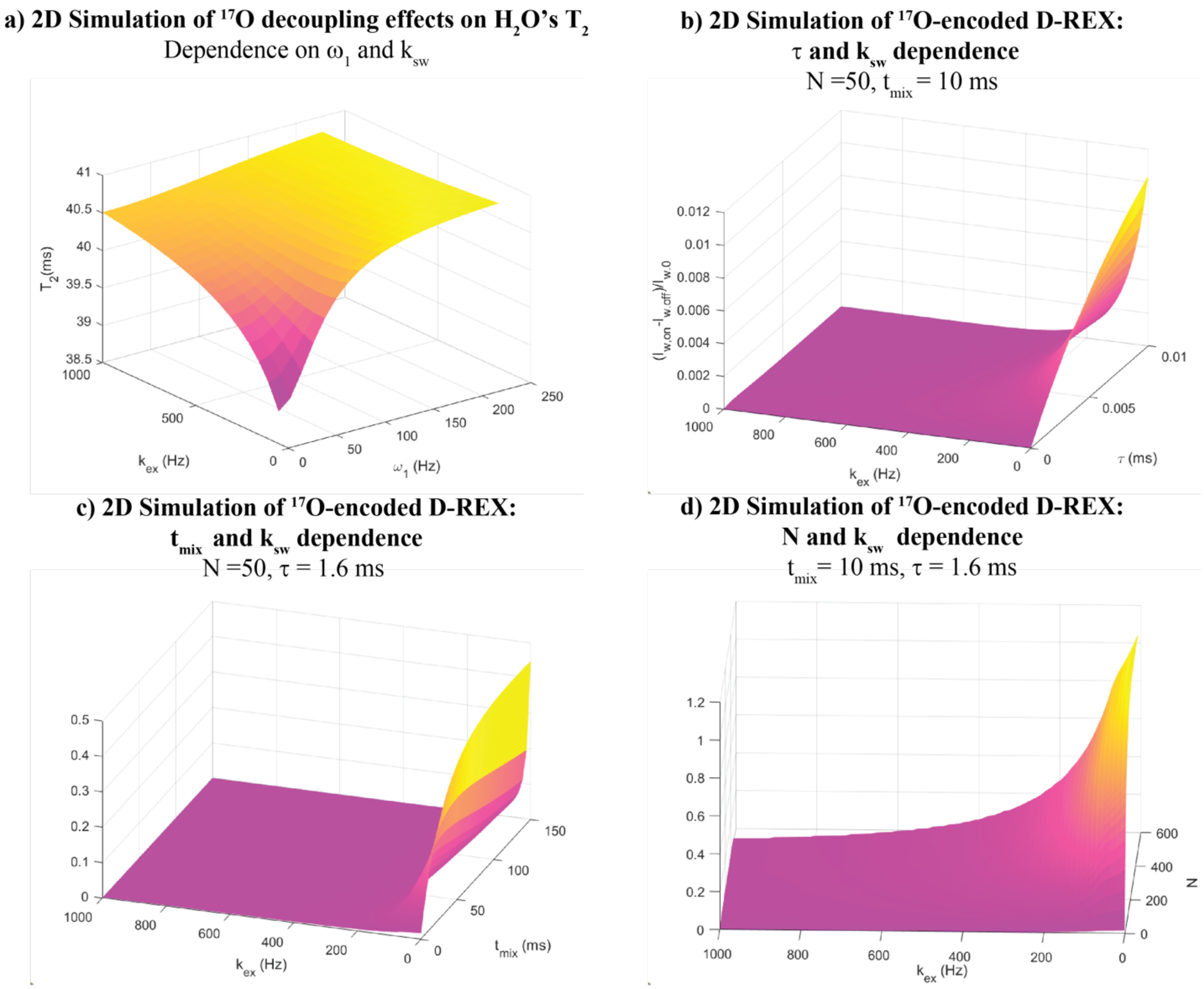

Figure S2: Maps of the analytical calculations of the effects of ^{17}O decoupling in natural abundance water, taking T_Q = 4.4 ms,[1] J = 91 Hz,[2] $T_{1,w}$ = 1.41 s, $T_{2,H}$ = 41 ms, with different exchange rates. a) Effect of decoupling into the average water T_2 with different decoupling fields, based on equation (3). b-d) Calculations of the ^{17}O-encoded D-REX effects based on equation (4) In each case the subsequent calculation took the optimal value of the latter for each k_{ex}. b) Calculation of the magnitude of the effect with regards to τ, with N = 50, and a t_{mix} of 10 ms. c) Calculation of the magnitude of the effect with regards to t_{mix}, with N = 50. d) Calculation of the magnitude of the effect with regards to N.

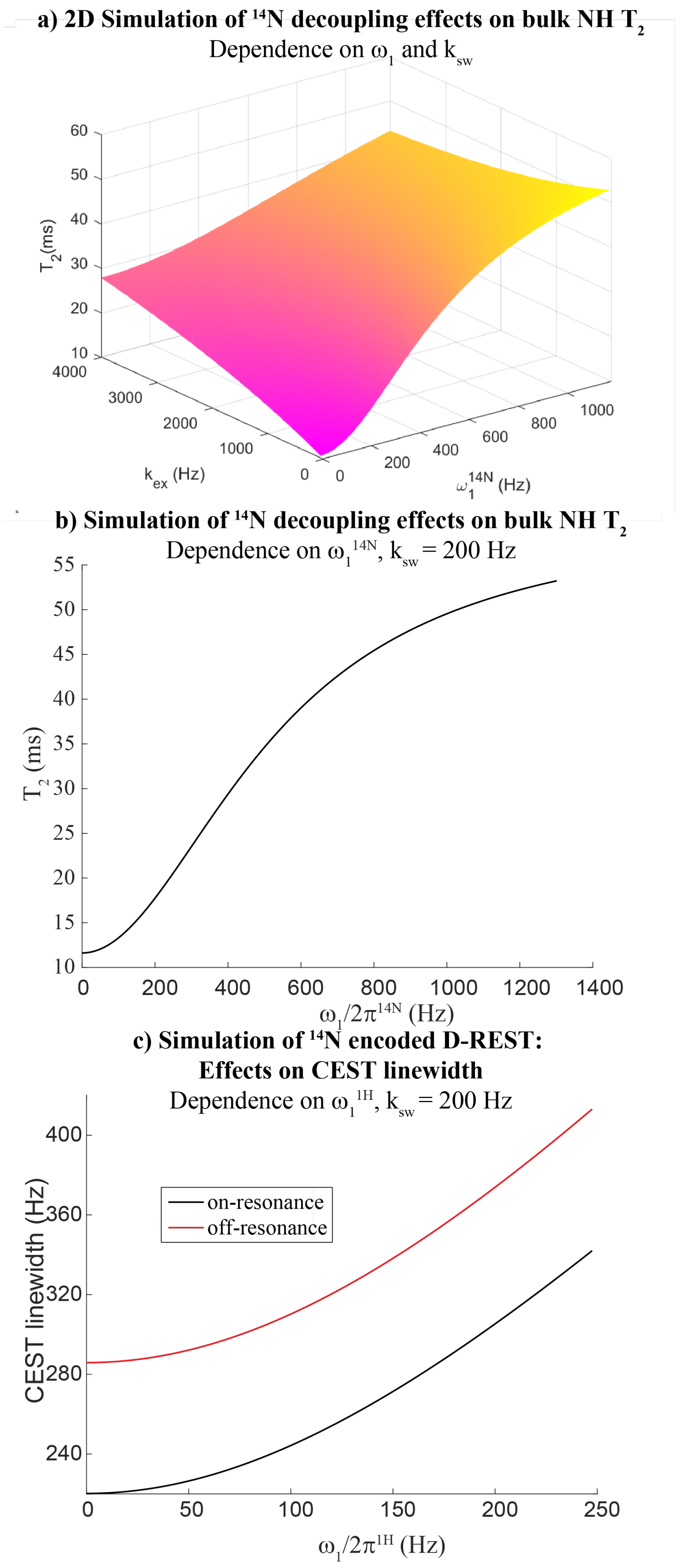
a) 2D Simulation of 14N decoupling effects on bulk NH T2
Dependence on ω1 and ksw
T2(ms)
60
50
40
30
20
10
4000
3000
2000
1000
0
kex (Hz)
0
200
400
600
800
1000
ω1 14N (Hz)
b) Simulation of 14N decoupling effects on bulk NH T2
Dependence on ω1 14N, ksw = 200 Hz
T2 (ms)
55
50
45
40
35
30
25
20
15
10
0
200
400
600
800
1000
1200
1400
ω1/2π 14N (Hz)
c) Simulation of 14N encoded D-REST:
Effects on CEST linewidth
Dependence on ω1 1H, ksw = 200 Hz
CEST linewidth (Hz)
400
360
320
280
240
on-resonance
off-resonance
0
50
100
150
200
250
ω1/2π 1H (Hz)

Figure S3: Analytical calculations of the effects of ^{14}N decoupling in dilute NH pair (20 mM), taking T_Q = 1 ms,[4] J = 62 Hz,[5], $T_{1,w}$ = 1.41 s, $T_{2,H}$ = 60 ms. a) Map of the effect of decoupling into the adjacent proton's T_2 with different decoupling fields and exchange rates. b) Calculation of the effect of decoupling into the adjacent proton's T_2 with different decoupling fields at k_{sw} = 200 Hz. Both these panels were based on equation (3). c) Calculations of the CEST linewidths with and without optimal decoupling based on equation (5), with k_{ex} = 200 Hz, with different CEST saturation fields. The difference between the resulting signals will be the observed signal in **^{14}**N encoded D-REST.

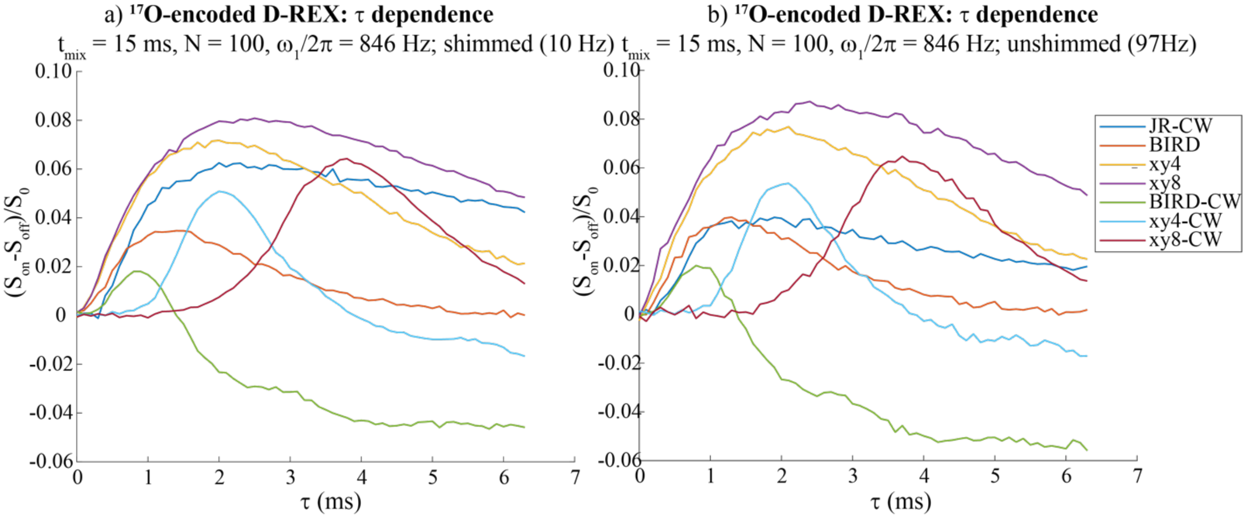

Figure S4: Comparison of the optimization of τ in ^{17}O-encoded D-REX conducted on a 50:50 mixture of water and DMSO-d$_6$, in which the water was enriched (with 25% ^{17}O -labeled water), in shimmed (a) and unshimmed (b) conditions. The magnet was unshimmed by varying the z1 coil values and the quality was assessed by the water's ^1H linewidth, 10 Hz for the shimmed data, and 97 Hz for the unshimmed ones. All the experiments were acquired in a 7 T field, at 24 °C. Versions of the different BIRD experiments, in which the π pulses were replaced by CW decoupling performed throughout the preparation period are also demonstrated. τ was optimization with N = 100, t_{mix} = 15 ms, decoupling RF field = 846 Hz. BIRD-based preparations were clearly more resilient to inhomogeneities than JR.

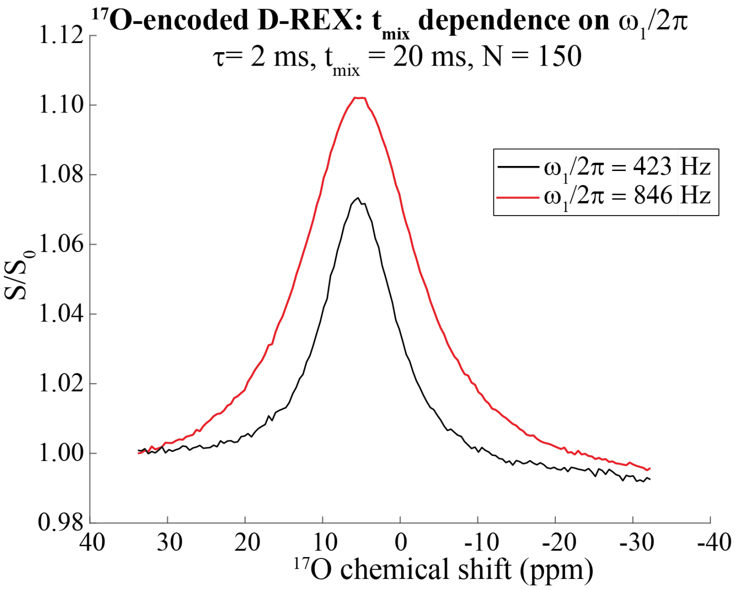

Figure S5: Indirect chemical shift mapping of the ^{17}O NMR spectrum for the sample introduced in Figure 3, the enriched 50:50 mixture of water and DMSO-d_6. Experiments were conducted in a similar fashion to Figure 5, though two different decoupling fields, 423 and 846 Hz were employed. This leads to an increase of the detected from ~7 to ~11 %, though also broadening the lines.

References

(1) Zhu, X. H.; Merkle, H.; Kwag, J. H.; Ugurbil, K.; Chen, W. 17O Relaxation Time and NMR Sensitivity of Cerebral Water and Their Field Dependence. *Magn. Reson. Med.* **2001**, *45*, 543–549.

(2) Burnett, L. J.; Zeltmann, A. H. 1H–17O Spin-spin Coupling Constant in Liquid Water. *J. Chem. Phys.* **1974**, *60*, 4636–4637.

(3) Meiboom, S. Nuclear Magnetic Resonance Study of the Proton Transfer in Water. *J. Chem. Phys.* **1961**, *34*, 375–388.

(4) Troganis, A. N.; Tsanaktsidis, C.; Gerothanassis, I. P. 14N NMR Relaxation Times of Several Protein Amino Acids in Aqueous Solution — Comparison with 17O NMR Data and Estimation of the Relative Hydration Numbers in the Cationic and Zwitterionic Forms. *J. Magn. Reson.* **2003**, *164*, 294–303.

(5) Witanowski, M.; Webb, G. A. *Nitrogen NMR*; Witanowski, M., Webb, G. A., Eds.; Springer US: Boston, MA, 1973.
